# Outcome analysis of ICSI assisted pregnancy using testicular sperm versus ejaculated sperm in man with severe oligozoospermia in the same ART cycle: A case report

**DOI:** 10.1097/MD.0000000000032833

**Published:** 2023-02-03

**Authors:** Liang Li, Shi bin Zhao

**Affiliations:** a Department of Reproductive Medicine, The Fourth Hospital of Hebei Medical University, Shijiazhuang, China.

**Keywords:** biologic outcomes, ejaculated sperm, ICSI, severe oligozoospermia, testicular sperm

## Abstract

Intracytoplasmic sperm injection (ICSI) has become the most common method for couples with male factor infertility, and source of sperm for the procedure have evolved over time. but few have examined testicular sperm extraction vs. ejaculated sperm use for severe oligozoospermia in the same assisted reproductive technology (ART) cycle.

**Patient concerns:**

Here, we evaluated the clinical outcomes after ICSI with testicular sperm or ejaculated in man with severe oligozoospermia in the same ART cycle. A couple who had failed the first ART cycle with ejaculated sperm, using the freshly ejaculated sperm and testicular sperm for ICSI during the second ART cycle by lack of enough sperm to fertilize in an ICSI attempt.

**Diagnoses:**

The patient was diagnosed with severe oligozoospermia, and routine semen analysis revealed sperm concentration is less than 2 million/mL.

**Interventions:**

The patient using testicular sperm versus ejaculated sperm with ICSI assisted pregnancy in the same ART cycle.

**Outcomes:**

We found that superior cleavage rate, number of embryos transferred and blastocyst rate with the use of testicular rather than ejaculated sperm-ICSI in the couple. The results described here suggest that use of testicular sperm may improve biologic outcomes, especially for couples with male-partner oligozoospermia who previous ICSI failures.

**Lessons:**

Our case report supported the efficacy of testicular sperm preference over ejaculated sperm for ICSI in men with severe male factor infertility. It is a paradigm shift concerning the use of ejaculated sperm as the preferable source of sperm for ICSI, add to the small amount of literature on testicular sperm extraction vs. ejaculated sperm use for severe oligozoospermia in the same ART cycle.

## 1. Introduction

Ejaculated sperm that have completed maturation during their passage through the male reproductive tract generally have better fertilization potential than testicular sperm.^[1]^ Accordingly, embryologists usually use of ejaculated sperm as the preferable source of sperm for intracytoplasmic injection (ICSI). The vast majority of patients with male factor infertility have sufficient sperm in the ejaculate for use with ICSI. Yet ejaculated sperm alone does not ensure adequate in ICSI outcomes, especially when used for severe oligozoospermia.

Several studies have addressed the potential benefit of testicular sperm compared with ejaculated sperm for ICSI in the treatment of oligozoospermia,^[2–4]^ but few studies have directly compared fertility outcomes after the use of ejaculated and testicular sperm for ICSI within patients in an assisted reproductive technology (ART) cycle. This case report described a couple who had previously failed the first ART cycle with ejaculated sperm, using the freshly ejaculated sperm and testicular sperm for ICSI during the second ART cycle by lack of enough sperm to fertilize in an ICSI attempt, we found that superior cleavage rate, number of embryos transferred and blastocyst rate with the use of testicular rather than ejaculated sperm-ICSI in the couple, we expect that this case report adds to opinions for using testicular sperm over ejaculated sperm, and it may provide superior ICSI outcomes for men with severe oligozoospermia. Patient has provided informed consent for publication of the case.

## 2. Case report

A 31-year-old male had a 2-year history of infertility after marriage. He exhibited a well-developed male phenotype. Physical examination showed a normal male habitus. Physical examinations were performed to detect varicocele and to measure testis volume. The left and right testes were each approximately 15 mL in volume. The patient did not present pathological varicocele. Routine semen analysis revealed sperm concentration is < 2 million/mL, the patient was thus diagnosed with severe oligozoospermia. Hormone analysis showed that serum follicle-stimulating hormone, luteinizing hormone, estradiol, prolactin, and testosterone levels were within the normal limits, which were listed in Table 1. Cytogenetic analysis showed that the presence of the sex-determining region on Yp, AZFa, AZFb, and AZFc regions on Yq, his karyotype was 46, XY. His wife karyotype was 46, XX, and her basal hormone profile was found to be within the normal limits, both sides of fallopian tube were passable was found on hysterosalpingography.

**Table 1:** Hormone analysis of the man with severe oligozoospermia.

	Levels	Normal range
FSH (mIU/mL)	3.02	1.5–12.4
LH (mIU/mL)	3.69	1.7–8.6
E2 (pg/mL)	28.96	25.78–60.66
PRL (ng/mL)	12.9	4.04–15.2
T (ng/mL)	5.08	2.49–8.36

E2 = estradiol, FSH = follicle-stimulating hormone, LH = luteinizing hormone, PRL = prolactin, T = testosterone.

Gonadotropin releasing hormone agonist starting from day 3 of the menstrual cycle were used for ovulation induction. Follicular development was monitored by the transvaginal ultrasonography. On the 10th day of the cycle, with follicles over 18 mm and estradiol levels of 3950 ng/mL, 250 µcg of human chorionic gonadotropin was administered. Transvaginal oocyte retrieval was performed 36 hours after human chorionic gonadotropin, yielding 22 oocytes, including 18 maturation oocytes, fresh sperm was obtained on the day of oocyte retrieva. Firstly, the sperm concentration was 2 million/mL, and no motile sperm was observed. His recent second sperm concentration was 4 million/mL, 1 motile sperm was seen per 7 to 8 high power field under high-times optic microscope. When ejaculated sperm was not enough to fertilize for ICSI, Testicular sperm retrieval was performed by testicular sperm extraction. Table 2 compares biologic outcomes of ICSI with extracted testicular sperm and ejaculated sperm. To prevent ovarian hyperstimulation syndrome, the patient underwent a frozen embryo transfer cycle 3 months later and achieved ongoing pregnancy, monozygotic twin pregnancies were diagnosed by ultrasound. Unfortunately, arrested intrauterine pregnancy occurred at 8 weeks.

**Table 2:** Comparison biologic outcomes of ICSI using testicular sperm versus fresh ejaculate sperm.

Testicular sperm-ICSI	Day0	Day 1	Day 2	Day 3	Day 5	Day6	
1	M II	2PN Z4	2PN	2PN			Disposal
2	M II	2PN Z4	3Cell III	5Cell IV			Disposal
3	M II	2PN Z3	pb	4Cell II	Stage 2 III	4BB	Transfer
4	M II	2PN Z3	2Cell I	4Cell I	Stage 2 III	Stage 2 IV	Disposal
5	M II	2PN Z4	2Cell I	4Cell I	4Cell IV		Disposal
6	M II	2PN Z3	2Cell I	7Cell II	7Cell IV		Disposal
7	M II	2PN Z3	4Cell I	7Cell II	Stage 2 III	Stage 2 IV	disposal
8	M II	2PN Z3	2Cell II	6Cell II	6Cell IV		Disposal
9	M II	0PN	pb	pb			Disposal
Ejaculate sperm-ICSI							
10	M II	0PN	pb	2Cell I			Disposal
11	M II	0PN	pb	2Cell I			Disposal
12	M II	0PN	pb	11Cell III	11Cell IV		Disposal
13	M II	0PN	pb	pb			Disposal
14	M II	0PN	pb	pb			Disposal
15	M II	0PN	pb	pb			Disposal
16	M II	0PN	pb	pb			Disposal
17	M II	0PN	pb	pb			Disposal
18	M II	0PN	pb	pb			Disposal

On D1, conventional grading of embryos was performed based on the Istanbul consensus in 2011. On D3, good quality embryos were defined as having equally sized, mononucleated blastomeres in a 3 dimensional tetrahedral arrangement, with 7–9 cells and < 10% fragmentation. On D5/6, Grading of blastocyst morphology were evaluated by Gardner.

ICSI = intracytoplasmic sperm injection.

## 3. Discussion

Recurrent in vitro fertilization (IVF) failure is a distressing situation, which is tough challenges for patients and doctors. Some of the unknown causes of recurrent IVF failure may be due to the man, the woman, or the couple. Of note, unexplained infertility has fallen mainly on women is always an emphasis. This case first report not sufficient sperm in the ejaculate for use with ICSI, the patient refused to re-ejaculate fresh sperm, and decided to undergo testicular sperm extraction-ICSI, the use of ejaculated sperm fertilizes half of the egg by ICSI, the rest of the egg was fertilized after ICSI with testicular sperm. When biologic outcomes were compared, the results of our study demonstrated that the proportion of cleavage, high quality embryo and blastocyst formation were lower in ejaculated sperm compared with testicular sperm from the same men with severe oligozoospermia.

Sperm DNA fragmentation (SDF) can negatively impact the fertility potential, including unexplained infertility, repeated intrauterine and IVF failure, and recurrent miscarriage.^[5,6]^ We postulated that differences in SDF between ejaculated and testicular sperm could be a reason for the differences seen in biologic outcomes. Indeed, testicular sperm have been suggested to have significantly lower levels of DNA damage compared with ejaculated sperm from the same individuals. DNA damage can be found in testicular sperm as a result of direct meiotic failure or early defective chromatin assembling at the spermatid stage, excessive oxidative stress are susceptible to damage sperm during passage through the male reproductive tract. Another potential explanation for this finding was semen samples in men with severe oligozoospermia undergoes extensive processing to be used in ICSI, which can increase oxidative stress and results in negative impact on ICSI outcomes. Therefore, the use of testicular sperm may be exempted from the oxidatively induced DNA damage taking place in the epididymis, becomes an favorable alternative in ART.

There is still an ongoing debate concerning the efficacy of testicular sperm over ejaculated sperm for ICSI in men with severe male factor infertility. Recent studies have reported improved embryo implantation rates, pregnancy rates and live birth rates, reduced miscarriage events using testicular sperm compared with ejaculated sperm in men with either severe oligospermia or cryptozoospermia,^[7,8]^ some studies confirmed that testicular in preference over ejaculated sperm might be advantageous for non-azoospermic men with high SDF,^[9]^ previous IVF/ICSI failures and aberrant mRNA protamine ratios.^[10]^ Conversely, other investigators did not show any differences in the ICSI outcomes using testicular versus ejaculated sperm in men with cryptozoospermia,^[11]^ patients with an elevated sperm chromatin structure assay-defined sperm DNA fragmentation and prior ICSI failures.^[12]^ These authors appeared to testicular sperm should not be recommended in preference over ejaculated sperm in men with severe oligozoospermia or cryptozoospermia.

In prior retrospective reports, the comparison of the different ovulation induction regimens cycle, there was some heterogeneity in terms of schemes, such as Medication schedule, trigger time, and oocyte quality. However, our data could not be confounded by some of the differences, we analysised the clinical outcomes after ICSI with testicular sperm and ejaculated during the same ovulation induction regimens cycle, hence our analysis was convincing. Limitations of this case include ejaculated sperm and testicular sperm failed to be tested by sperm chromatin structure assay, because the sperm DNA assay requires a minimum of 1 million cells/mL,^[13]^ and we failed to persuade the couple to detect villi tissue chromosome. As we know, a higher risk of main obstetric complications in monozygotic twin pregnancies. It is an undeniable fact that miscarriage, preterm delivery, low birth weight, growth restriction, developmental anomalies, preeclampsia, perinatal morbidity, and mortality are all increasing.^[14]^ Time-lapse monitoring may provide objective and accurate information for early embryo development in vitro. It is an effective option to understand the characteristics of embryonic preimplantation development of testicular versus ejaculated spermatozoa in ICSI. It is regrettable that we fail to store and record data with Time-lapse monitoring.

In conclusion, we report a rare case of testicular sperm versus ejaculated sperm use for severe oligozoospermia in the same ART cycle. Testicular sperm maybe preferable source of sperm for ICSI in cases of infertility associated with severe oligozoospermia, it is a new strategy to overcome infertility in men with oligozoospermia.

## Author contributions

**Conceptualization:** Shi bin Zhao.

**Data curation:** Liang Li, Shi bin Zhao.

**Formal analysis:** Liang Li.

**Funding acquisition:** Shi bin Zhao.

**Writing – original draft:** Liang Li, Shi bin Zhao.

**Writing – review & editing:** Liang Li, Shi bin Zhao.
